# Carbolic Acid Dangerous Every Way

**Published:** 1899-09

**Authors:** Gregory Doyle


### Carbolic Acid Dangerous Every Way.

It is well known that this acid in its concentrated form is a rapidly fatal poison when taken internally. Many, however, ar- not aware that its external application, even when diluted with water or in the form of an ointment, is extremely dangerous. Local gangrene is the penalty of too strong an application, and often of a weak application too long applied. The facility with which it is absorbed by the skin and the rapidity and virulence with which it affects the nervous system and internal organs, especially the kidneys, classes it among the most dangerous drugs known. Its indiscriminate use has become quite a domestic fad, and it is now used in the family for almost every ailment from a soft corn to a hard headache. As it is extremely dangerous to health and life, it should be used with the greatest caution, and then only with the advice of some one thoroughly familiar with its dangers and possibilities.—*Dr. Gregory Doyle.*

